# The Molecular Basis of Amino Acids Sensing

**DOI:** 10.1002/advs.202501889

**Published:** 2025-05-24

**Authors:** Cong Jiang, Xiao Tan, Jiali Jin, Ping Wang

**Affiliations:** ^1^ Shanghai Tenth People's Hospital School of Medicine Tongji University Cancer Center Tongji University Shanghai 200092 China

**Keywords:** amino acid sensing, amino acid sensor, nutrient stress, mTORC1

## Abstract

Amino acids are organic compounds that serve as the building blocks of proteins and peptides. Additionally, they function as bioactive molecules that play important roles in metabolic regulation and signal transduction. The ability of cells to sense fluctuations in intracellular and extracellular amino acid levels is vital for effectively regulating protein synthesis and catabolism, maintaining homeostasis, adapting to diverse nutritional environments and influencing cell fate decision. In this review, the recent molecular insights into amino acids sensing are discussed, along with the different sensing mechanisms in distinct organisms.

## Graphical abstract

Amino acids are essential as protein building blocks and signaling molecules, enabling metabolic regulation. Cells sense amino acid levels to control protein synthesis, maintain homeostasis, and adapt to nutritional changes. This review explores recent advances in amino acid sensing mechanisms across organisms, highlighting their roles in cellular adaptation and fate decisions.

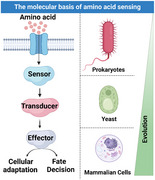

## 1 Introduction

Amino acids, along with glucose and lipids, are one of the three macronutrients essential for generating energy and providing biomass.^[^ ^1^, ^2^, ^3^ ^]^ Each amino acid consists of a central carbon atom connected to an amino group (NH2), a carboxyl group (COOH), a hydrogen atom, and a variable side chain (R group) that determines their unique properties and distinguish them from other amino acids.^[^ ^3^ ^]^ Based on their essentiality, amino acids are traditionally categorized into essential amino acids (EAAs) or nonessential amino acids (NEAAs). EAAs, which comprise leucine, isoleucine, valine, tryptophan, phenylalanine, lysine, methionine, threonine, and histidine, cannot be synthesized by the organism on its own and must be acquired through the diet.^[^ ^3^ ^]^ Nonessential amino acids (NEAAs), which include alanine, asparagine, aspartic acid, glutamic acid, serine can be obtained directly from diet.^[^ ^3^ ^]^ The other amino acids, including arginine, cysteine, glutamine, glycine, proline, and tyrosine, are conditionally essential. For healthy individuals, the body's de novo synthesis of these amino acids is sufficient to meet physiological needs. However, during times of stress or illness, it becomes necessary to obtain these amino acids through dietary intake.^[^ ^4^ ^]^

Amino acids are primarily recognized for their role in enhancing protein synthesis and reducing protein breakdown.^[^ ^5^ ^]^ Additionally, during conditions of excess amino acids, prolonged starvation, or hypoglycemia, the deamination of amino acids results in carbon skeletons that can be converted into intermediates feeding into gluconeogenesis, ketogenesis, and fatty acid synthesis.^[^ ^5^ ^]^ Moreover, amino acids can function as the foundational blocks for neurotransmitters or be involved in hormone synthesis, influencing mood, cognition, and behavior, as well as contributing to metabolic regulation.^[^ ^1^ ^]^ Furthermore, amino acids such as cysteine and methionine are vital for detoxification, producing glutathione which neutralizes reactive oxygen species and facilitates the excretion of toxins through bile or urine.^[^ ^1^, ^2^, ^3^ ^]^ To coordinate the amino acid availability for these varied biological responses, cells must be able to sense the amino acids levels in their environment. The sensing of amino acid encompasses the direct binding of amino acids or their derivatives to their specific sensors. Furthermore, sensors should adapt their behavior to trigger downstream effects in response to the fluctuating levels of amino acids or their surrogates within their physiological or pathological ranges. The precise sensing of amino acid levels and their intermediates is essential for managing protein anabolism and catabolism, maintaining cellular and organismal metabolic homeostasis and adapting to diverse environmental conditions. Both prokaryotic and eukaryotic organisms have evolved sophisticated extracellular and intracellular amino acid sensing pathways, though the underlying mechanisms differ significantly. In prokaryotes, amino acid sensing is primarily mediated through post‐translational modifications. In contrast, eukaryotes employ a combination of conserved pathways—such as GCN2 and mTORC1—and lineage‐specific mechanisms, like those found in yeast, to detect and respond to amino acid availability. In this review, we explore the varied amino acid sensing mechanisms across different organisms and discuss how these mechanisms have evolved to accommodate specific needs and diverse nutritional environments.

## 2 Amino Acid Sensing in Prokaryotes

Bacteria have evolved numerous fascinating mechanisms, utilizing single‐pass transmembrane chemoreceptors or enzymes to sense various amino acids and their metabolites, enabling adaptation to different living environments. It is worth noting that post‐translation modifications, including phosphorylation, adenylation, and methylation, play important role in regulating bacterial amino acid sensing.

### 2.1 Extracellular Amino Acid Sensing in Prokaryotes

**Figure 1:**
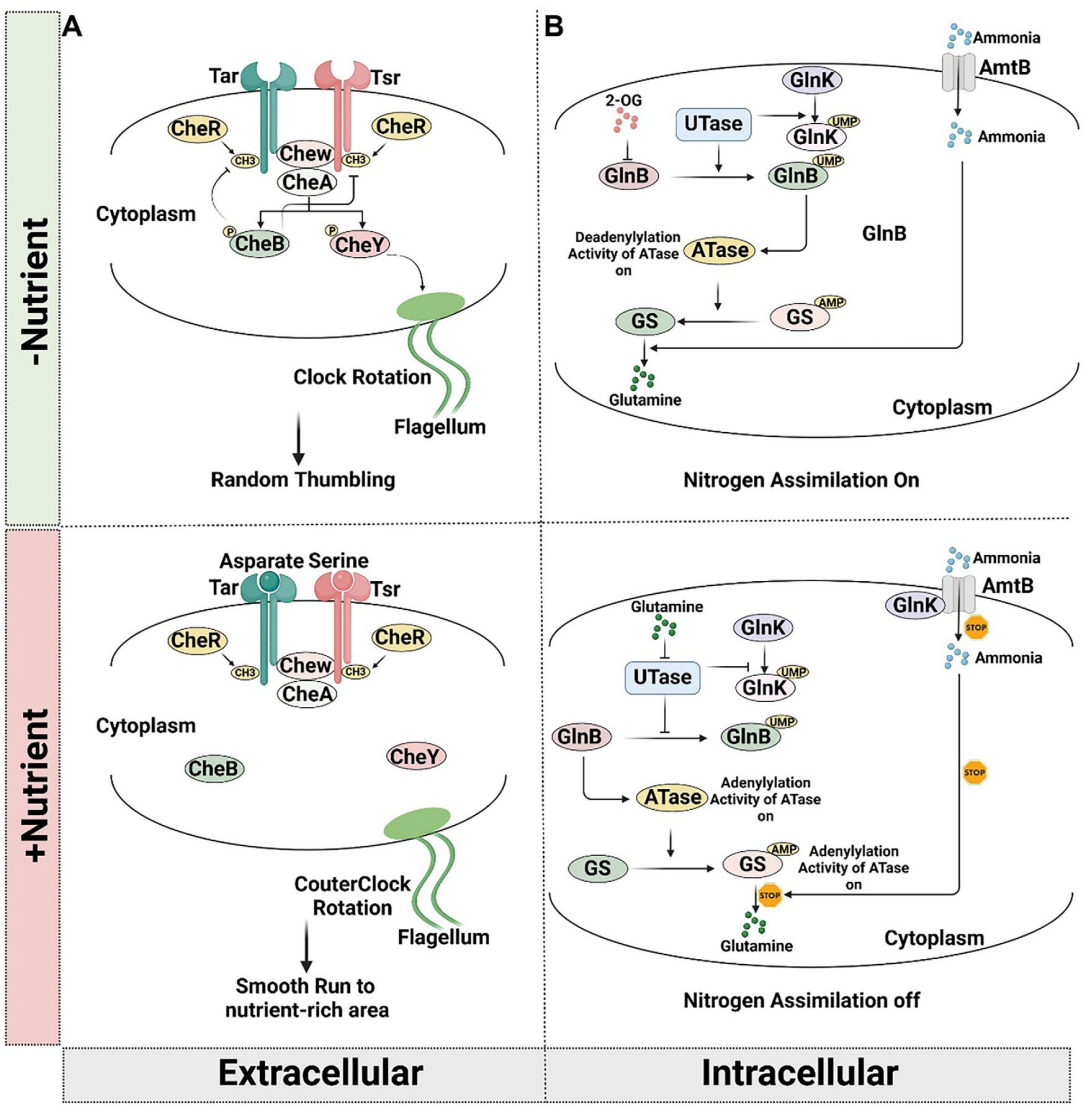
Amino acid sensing pathway in prokaryotes. A) The extracellular amino acid sensing pathway in prokaryotes. In the absence of attractants (aspartate/serine), CheA autophosphorylates, activating CheY (promoting tumbling) and CheB (reducing receptor methylation to drive adaptation). When attractants bind, CheA activity is inhibited, decreasing CheY‐P (favoring smooth swimming) and CheB activity, while CheR methylates receptors to restore CheA signaling (enabling adaptation). B) Intracellular amino acid sensing pathway in prokaryotes. Prokaryotes sense nitrogen availability through metabolites: glutamine (excess) and α‐ketoglutarate (α‐KG, scarcity). Under nitrogen‐rich conditions, glutamine inhibits UTase, preventing PII protein (GlnB) uridylylation. Unmodified GlnB activates ATase, adenylylating and inactivating glutamine synthetase (GS), suppressing nitrogen assimilation. During nitrogen limitation, α‐KG binds GlnB, blocking ATase activation, leaving GS active to enhance assimilation. The PII protein GlnK regulates ammonium uptake via AmtB. Under scarcity, uridylylated GlnK dissociates from AmtB, maximizing ammonium import. Upon sudden ammonium increase, deuridylylated GlnK binds AmtB, inhibiting transport.

Bacteria exhibit chemotaxis, a process in which they swim towards nutrient‐rich areas by sensing chemical gradients and adjusting their flagellar motion to navigate toward these concentrations, optimizing their growth and survival.^[^ ^6^, ^7^, ^8^ ^]^ *E. coli* is equipped with five dimeric, single‐pass transmembrane chemoreceptors—Tar, Tsr, Tap, Trg, and Aer—that each serve as specific sensors for various nutrients.^[^ ^9^, ^10^ ^]^ Among these five chemoreceptors, Tar and Tsr are the most prevalent, with Tar binding to aspartate and Tsr binding to serine, each recognizing specific amino acids.^[^ ^9^, ^10^ ^]^ (**Figure** **1**A). Of note, these chemoreceptors are highly sensitive, able to detect ligand concentrations across a broad spectrum,^[^ ^11^ ^]^ allowing cells to perceive amino acids even in extremely dilute environments.

CheA is a homodimeric histidine kinase that constitutively associates with Tar and Tsr and its adaptor protein, CheW.^[^ ^12^, ^13^, ^14^, ^15^ ^]^ The signaling states of Tar and Tsr are regulated by the actions of the methyltransferase CheR and the methylesterase CheB.^[^ ^16^ ^]^ In the absence of aspartate or serine, Tar or Tsr stimulates CheA, which subsequently phosphorylates and activates two diffusible effectors. CheY, which causes the flagellar motor to rotate clockwise, resulting in random tumbling,^[^ ^14^ ^]^ and CheB, which demethylase the chemoreceptor and reduces its capacity to activate CheA, promoting adaptation to amino acid deficient condition.^[^ ^14^, ^16^ ^]^ Conversely, the presence of aspartate or serine leads to their binding with Tar or Tsr, which triggers a conformational change that inhibits CheA activation and subsequently reduces the phosphorylation of CheY, allowing bacteria to orient their movements toward regions with higher concentrations of nutrients.^[^ ^12^, ^14^, ^15^, ^17^ ^]^ Meanwhile, CheB's demethylation activity is suppressed while methyltransferase CheR is constitutively active, leading to increased methylation of Tar or Tsr.^[^ ^12^, ^14^, ^15^, ^17^ ^]^ This methylation boosts CheR's capacity to activate CheA, aiding adaptation to elevated levels of amino acids.^[^ ^18^, ^19^ ^]^ Thus, the methylation status of Tar or Tsr helps *E. coli* adapt to the existing environment, allowing the system to return to the pre‐stimulus level and remain ready to react to subsequent environmental shifts.

### 2.2 Intracellular Amino Acids Sensing in Prokaryotes

Besides the aforementioned extracellular amino acid sensing system, prokaryotes also have an internal pathway for sensing nitrogen deficiency. When nitrogen is scarce, prokaryotes boost their nitrogen assimilation by creating nitrogen‐containing molecules like amino acids from inorganic nitrogen sources in the environment. This process is facilitated by the enzymes glutamine synthetase (GS) and glutamate synthase (GOGAT), which together convert α‐ketoglutarate (α ‐KG), ammonium, and ATP into glutamine.^[^ ^20^, ^21^ ^]^ The sensing process is facilitated by two distinct metabolites: glutamine, indicative of nitrogen excess, and α‐ketoglutarate, indicative of nitrogen shortage. These are recognized respectively by the uridylyltransferase (UTase) enzyme and the PII superfamily of proteins, namely GlnB^[^ ^20^ ^]^ (Figure 1B). When there is sufficient nitrogen, glutamine binds to UTase and inhibits its ability to uridylylate PII proteins, GlnB.^[^ ^22^, ^23^ ^]^ The unuridylylated GlnB allosterically activates the adenylylation activity of ATase, which results in the adenylylation of GS, rendering GS inactive and suppress nitrogen assimilation.^[^ ^22^, ^23^ ^]^ In the absence of nitrogen, α‐ketoglutarate (α‐KG), a precursor for nitrogen assimilation, accumulates. α‐KG then binds to the unuridylylated GlnB, which fails to activate the adenylylation reaction of ATase.^[^ ^24^, ^25^, ^26^, ^27^ ^]^ This leads to the accumulation of the unadenylylated and active form of GS, thereby enhancing nitrogen assimilation.

Additionally, GlnK, another PII protein, plays a crucial role in sensing nitrogen availability by regulating the activity of AmtB, an ammonia transporter, in a way similar to GlnB.^[^ ^28^, ^29^ ^]^ Under conditions of nitrogen scarcity, GlnK is primarily in its fully uridylylated state and does not strongly associate with the membrane.^[^ ^28^, ^29^ ^]^ Consequently, AmtB remains active and efficiently scavenges ammonium from the external environment. When extracellular ammonium levels suddenly increase, GlnK rapidly deuridylylates and binds tightly to AmtB in the inner membrane, thereby reducing AmtB's activity and decreasing ammonium transport into the cell.^[^ ^28^, ^29^ ^]^

Therefore, instead of directly detecting amino acids like chemoreceptors, prokaryotes use an alternative pathway that senses amino acid intermediates to respond to their availability. This sensing pathway enables them to swiftly adapt to changes in the internal nutritional environment, meeting their needs for energy and biomass. Moreover, this sensing mechanism confer specificity to this process, differentiating it from other ammonium‐dependent reactions.

## 3 Amino Acid Sensing in Eukaryotes

Eukaryotes have evolved an amino acid sensing system that connects both the extracellular and intracellular availability of amino acid to various internal processes, ranging from gene expression that dictates nutrient uptake and utilization for optimal growth to decisions about cell fate. Yeast has developed a unique extracellular amino acid sensing system, the Ssy1‐Ptr3‐Ssy5 (SPS) pathway, which is not found in other eukaryotes. In contrast, the intracellular amino acid sensing pathways mediated by GCN2 and mTORC1 are conserved across species from yeast to mammals. In this section, we will explore both the yeast‐specific amino acid sensing pathway and the conserved amino acid sensing pathways across species.

### 3.1 Extracellular Amino Acid Sensing System Unique in Yeast: SPS Pathway

**Figure 2:**
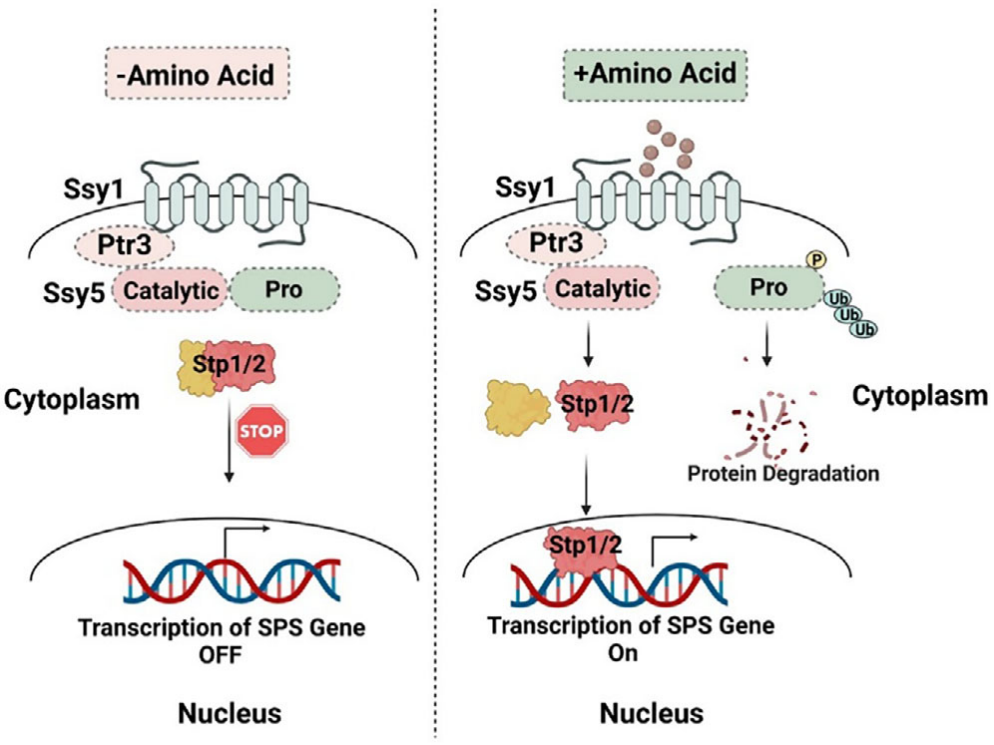
Amino acid sensing pathway unique in yeast. Yeast senses extracellular amino acids via the SPS complex (Ssy1‐Ptr3‐Ssy5). Ssy1, a transporter‐like sensor (“transceptor”), detects amino acids and triggers Ptr3‐mediated activation of the endoprotease Ssy5. Active Ssy5 cleaves transcription factors Stp1/2, enabling nuclear translocation and induction of amino acid metabolism/transport genes.

Yeast utilizes transmembrane proteins to sense amino acids, similar to bacteria. However, unlike bacterial chemoreceptors, these yeast sensors are homologous to nutrient transporters and are sometimes referred to as transceptors. The transmembrane protein Ssy1 forms a complex with Ptr3 and Ssy5, known as the SPS pathway, to convey extracellular amino acid availability to downstream transcription of amino acid transporter and metabolic genes.^[^ ^30^, ^31^ ^]^ This system enables yeast to regulate the uptake and utilization of amino acids, optimizing growth based on available resources (**Figure** **2**).

Ssy1, which resembles a transporter protein but lacks transporter activity, uses its N‐terminal extension to relay amino acid availability to downstream signaling components.^[^ ^31^, ^32^, ^33^, ^34^, ^35^ ^]^ Ssy5, an endoprotease, contains an inhibitory prodomain and a catalytic domain.^[^ ^36^, ^37^, ^38^ ^]^ In the presence of abundant amino acids, they bind to Ssy1, causing a conformational change. Ptr3 serves as an adaptor, transferring this change to Ssy5 and aligning the prodomain of Ssy5 with its kinase, which leads to the phosphorylation and ubiquitin‐mediated degradation of the prodomain. This action ultimately lifts the inhibition on Ssy5's catalytic domain, allowing it to cleave and activate the transcription factors Stp1 and Stp2, facilitating their nuclear translocation to activate specific genes.^[^ ^36^, ^37^, ^38^ ^]^ There are two key pieces of evidence indicating that the SPS pathway directly senses amino acids in yeast. Firstly, mutations in specific amino acids change the SPS complex's sensitivity to extracellular amino acids.^[^ ^39^ ^]^ Second, mutations in Ptr3 or Ssy1 lead to increased accumulation of basic amino acids in vacuoles.^[^ ^31^ ^]^ This indicates that yeast store and utilize amino acids within vacuoles to meet their energy and biomass requirements in the absence extracellular amino acids signaling pathway, thereby maintaining homeostasis even in the absence of external amino acid supply. This adaptation is essential for yeast to survive in amino acid‐deprived conditions.

### 3.2 Amino Acids Sensing Pathways Conserved from Yeast to Mammals

Alongside the extracellular amino acid sensing pathway mediated by the SPS pathway, eukaryotes have evolved parallel intracellular pathways, namely the GCN2 and mTORC1 pathways.^[^ ^40^ ^]^ Throughout evolution, there has been crosstalk between these pathways, occurring in different directions. In yeast, GCN2 functions downstream of mTORC1, whereas in mammals, it operates upstream of mTORC1.^[^ ^40^ ^]^

#### 3.2.1 GCN2‐Mediated Amino Acid Sensing Pathway

**Figure 3:**
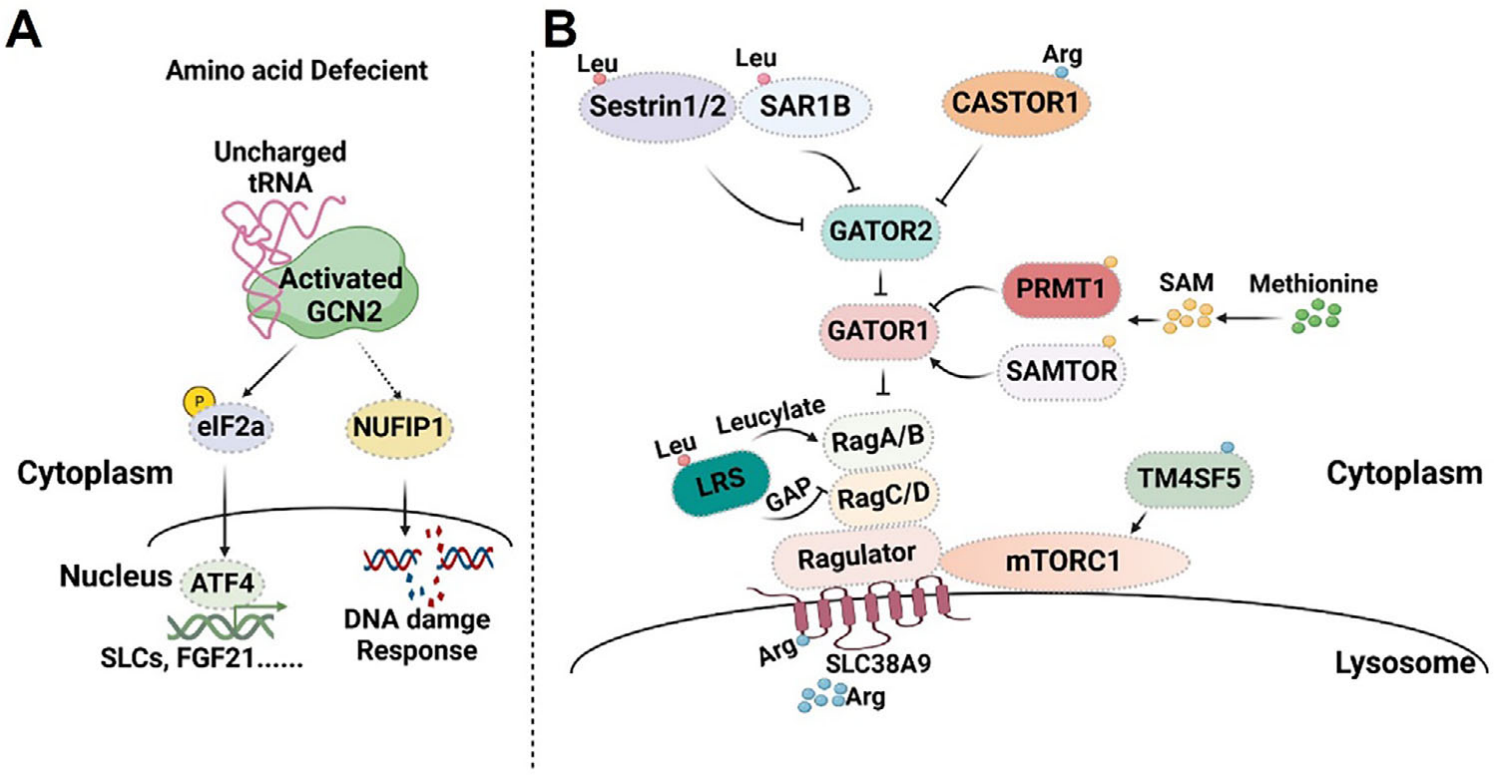
GCN2 and mTORC1‐mediated amino acid sensing in eukaryotes. A) GCN2‐mediated amino acid sensing. B) mTORC1 mediated amino acid sensing.

General Control Non‐derepressible 2 (GCN2) acts as a sensor for amino acid deficiency. When amino acids are absent, specific aminoacyl tRNA synthetases (aaRSs) cannot attach amino acids to their corresponding tRNAs, resulting in an accumulation of uncharged tRNAs^[^ ^41^, ^42^, ^43^ ^]^ (**Figure** **3**A). These uncharged tRNAs bind to GCN2, causing a conformational shift and autophosphorylation that allows GCN2 to phosphorylate and inhibit its downstream target, eIF2. This phosphorylation restricts the efficient initiation of translation for most mRNAs, acting as a checkpoint in the translation process.^[^ ^41^, ^42^, ^43^ ^]^ The activation of GCN2 also triggers the upregulation of transcription factor GCN4 in yeast and ATF4 in mammals by promoting their translation. This upregulation sets off a cascade of transcriptional regulators that stimulate a gene expression program impacting apoptosis, autophagy, and amino acid metabolism. This program includes enhancing specific aminoacyl tRNA synthetases and amino acid transporters, thereby increasing amino acid uptake and meeting protein synthesis demands as an adaptation to nutrient deficiency.^[^ ^44^, ^45^, ^46^, ^47^, ^48^ ^]^ Notably, cancer cells respond to shortages of specific amino acids—glutamine, arginine, methionine, or lysine—by triggering the GCN2‐ATF4‐REDD1 pathway. This signaling cascade ultimately activates AKT, enhancing cell survival during periods of amino acid scarcity.^[^ ^49^ ^]^ GCN2 can bind to any uncharged tRNAs without showing specificity for any particular amino acid. Considering that the absence of any amino acid can potentially disrupt peptide chain synthesis during protein production, the GCN2‐mediated amino acid sensing pathway serves as an effective model to prevent such failures in peptide chain synthesis.

In addition, the research group led by Lei et al. reported that low protein diet can reduce the expression of nuclear fragile X mental retardation‐interacting protein (NUFIP1) in a GCN2 dependent manner, which mitigates NUFIP1‐mediated DNA damage response (DDR) and subsequently induces DNA damage and activates necroptosis in intestinal cells, leading to bowel inflammation.^[^ ^50^ ^]^ The GCN2‐NUFIP1‐DDR mediated amino acid sensing pathway is critical to maintain intestinal homeostasis via dictating the accuracy of DNA replication to meet the needs for its rapid cell proliferation under amino acid sufficient condition. When lacking amino acids, this sensing pathway is interrupted and eventually leads to DNA damage and RIPK3‐mediated necroptosis, establishing a functional connection between the amino acid availability and cell fate decision, as well as inflammation at the tissue level.

##### mTORC1‐Mediated Amino Acid Sensing Pathway

The mTORC1 pathway is another crucial signaling mechanism that senses intracellular amino acid availability and has evolved in parallel with the GCN2 pathway. It is a serine/threonine protein kinase complex that includes multiple components: mTOR, which functions as the catalytic core; mammalian lethal with SEC13 protein 8 (mLST8); regulatory‐associated protein of mTOR (RAPTOR); proline‐rich AKT substrate 40 kDa (PRAS40); and DEP domain‐containing mTOR interacting protein (DEPTOR). mTORC1 responds to diverse environmental signals such as amino acids, glucose, oxygen, cholesterol, and stress.^[^ ^51^, ^52^, ^53^, ^54^ ^]^ It regulates both anabolic and catabolic metabolic processes, including the synthesis of proteins, lipids, and nucleotides, and the process of autophagy, by phosphorylating downstream targets.^[^ ^51^, ^52^, ^53^, ^54^ ^]^ This activity is essential to preserve the cellular and organismal homeostasis. Here, we mainly introduce amino acids sensing mechanism of mTORC1 signaling, a process highly conserved from yeast to mammals (Figure 3B).

Two distinct small GTPase localized on the lysosomal surface integrates inputs from amino acids and growth factors to the fully activation of mTORC1 at the lysosomal surface. One component is Rheb,^[^ ^55^, ^56^ ^]^ which dictates the kinase activity of mTORC1, primarily integrating inputs from growth factors. Another key element is Rag GTPase, which forms obligate heterodimers, consisting of either RagA or RagB paired with RagC or RagD.^[^ ^57^, ^58^ ^]^ The nucleotide status of this complex is essential for the recruitment of mTORC1 to the lysosome, where it is activated by Rheb.^[^ ^59^ ^]^ The presence of amino acids primarily signals through the Rag GTPase by altering its nucleotide status. In the presence of amino acids, the nucleotide loading of this complex is RagA/B^GTP^‐RagC/D^GDP^, indicating an active state. Conversely, in the absence of amino acids, the nucleotide configuration shifts to RagA/B^GDP^‐RagC/D^GTP^, rendering the complex inactive.^[^ ^57^, ^58^ ^]^ There are also amino acid‐sensing mechanisms independent of Rag GTPase. In these pathways, Arf1 is essential for glutamine‐mediated activation of mTORC1m,^[^ ^60^ ^]^ and Rab1a plays a critical role in recruiting mTORC1 to the Golgi apparatus.^[^ ^61^ ^]^

Multiple signaling complexes dictates the nucleotide state of Rag GTPase under diverse amino acids conditions. Among these complexes, Ragulator and vacuolar ATPase (v‐ATPase) play crucial roles in amino acid sensing of mTORC1.^[^ ^62^, ^63^, ^64^, ^65^ ^]^ Ragulator, composed of LAMTOR1 (p18), LAMTOR2 (p14), LAMTOR3 (MP1), LAMTOR4 (C1ORF59), and LAMTOR5 (HBXIP), along with v‐ATPase, regulates the lysosomal localization and the activation of Rag GTPase by amino acids derived from lysosomes.^[^ ^62^, ^63^, ^64^, ^65^, ^66^ ^]^ Two distinct GTPase‐activating protein (GAP) complexes, promoting GTP hydrolysis of RagA/B/C/D complex, with GATOR1(NPRL2/NPRL3/DEPDC5) acting on RagA/B and Folliculin‐FNIP2(FLCN/FNIP1/FNIP2) on RagC/D.^[^ ^67^, ^68^ ^]^ KICSTOR(KPTN/ITFG2/C12orf66/SZT2) and ILF3 are critical for the lysosomal positioning of GATOR1 complexes.^[^ ^69^, ^70^, ^71^ ^]^ The GAP activity of GATOR1 in counteracted by another pentameric complex known as GATOR2(WDR59, WDR24, Mios, SEC13, and SEH1L).^[^ ^68^, ^72^, ^73^ ^]^

Although key components of mTORC1's amino acid sensing branch have been identified, the direct amino acid sensor within mTORC1 signaling remained elusive until Sestrin2 was discovered to act as a leucine sensor upstream of mTORC1. This field has seen rapid advancements in recent years, identifying both cytosolic and lysosomal sensors for various amino acids. In this section, we will explore the identified amino acid sensors and their associated sensing mechanisms known to date.

##### Leucine Sensor of mTORC1 Signaling

Sestrin2 and SAR1B are cytosolic leucine sensors that converge on different subunits of the GATOR2 complex by recognizing distinct structural forms of leucine.^[^ ^74^, ^75^, ^76^ ^]^ Without leucine, Sestrin2 and SAR1B bind to and inhibit GATOR2, thus releasing GATOR2's inhibitory effect on GATOR1.^[^ ^74^, ^75^, ^76^ ^]^ When leucine is present, it binds to Sestrin2 and SAR1B, causing them to dissociate from the GATOR2 complex, enabling GATOR2 to resume its inhibitory action on GATOR1.^[^ ^74^, ^75^, ^76^ ^]^

However, these two proteins exhibit distinct leucine sensitivities and tissue distributions, functioning independently to relay leucine availability to mTORC1. Sestrin2 binds leucine with a *K*_d_ of ≈20 µm, recognizing both the amino and carboxyl groups, whereas SAR1B exhibits a higher affinity (*K*_d_ ≈ 2 µm) by interacting with the amino group and side chain of leucine. This difference enables a two‐phase mTORC1 activation mechanism, driven by the sequential dissociation of SAR1B and Sestrin2 from GATOR2. Furthermore, SAR1B is predominantly expressed in skeletal muscle, while Sestrin2 is highly abundant in adipose tissue, suggesting tissue‐specific leucine sensing by mTORC1. Moreover, Sestrin1 and Sestrin2 have been reported to regulate mTORC1 leucine sensing both in vitro and in vivo,^[^ ^77^ ^]^ establishing them as physiological leucine sensors. In contrast, Sestrin3–another homolog of Sestrin2–constitutively binds to GATOR2 regardless of leucine concentration. Whether Sestrin3 is regulated by other signals remains to be determined.^[^ ^74^, ^75^ ^]^ Additionally, leucyl‐tRNA synthetase (LRS) has been proposed as another leucine sensor, either acting as the GAP for RagC/D or directly leucylating RagA/B to activate mTORC1.^[^ ^78^, ^79^ ^]^ The affinity of these sensors for leucine varies, with Sestrin2 having an affinity of about 20 µm,^[^ ^75^ ^]^ while LRS shows an affinity of approximately 40 µm.^[^ ^80^ ^]^ Under conditions where leucine is abundant, the leucine‐laden LRS forms a complex with RagD, promoting GTP hydrolysis and thereby activating mTORC1.^[^ ^81^ ^]^ Structural studies identify two operational modes for LARS1: “sensing‐on” and “sensing‐off.” In the sensing‐on mode, LARS1 engages with RagD to trigger mTORC1 activation. Upon the synthesis of Leu‐AMP from leucine and ATP, LARS1 transitions to the sensing‐off mode, detaching from RagD, which results in the deactivation of mTORC1. Subsequently, LARS1 is available to bind to tRNA, facilitating the production of Leu‐tRNA^Leu^ for protein synthesis. In contrast to human cells, it has been proposed that in yeast, leucyl‐tRNA synthetase (LRS) regulates mTORC1 through its interaction with Gtr1 (the yeast equivalent of RagA/B) and demonstrates GEF‐like activity by enhancing GTP loading on RagA.^[^ ^82^ ^]^ Supporting this, LRS was observed leucylating RagA at K142 and RagB at K203 in human cells, thereby enhancing GTP loading on RagA/B via inference its interaction with Ragulator and GATOR1.^[^ ^79^ ^]^ These diverse observations introduce new questions and complexities into the existing model of leucine sensing and mTORC1 activation. How these various sensing mechanisms work together to relay leucine availability to mTORC1 remains unclear.

##### Arginine Sensor of mTORC1 Signaling

CASTOR1 and SLC38A9 have been proposed as arginine sensors in mTORC1 signaling.^[^ ^62^, ^83^, ^84^, ^85^, ^86^, ^87^, ^88^ ^]^ CASTOR1, a cytosolic arginine sensor, converges on the GATOR2 complex, while SLC38A9, a lysosomal arginine sensor, functions by interacting with the Rag GTPase–Ragulator‐v‐ATPase complex. Like Sestrin2, CASTOR1 binds to and inhibits GATOR2 when arginine is absent, but it detaches upon arginine binding, thus enabling the activation of mTORC1.^[^ ^83^, ^84^ ^]^ Unlike CASTOR1, CASTOR2 forms a stable complex with GATOR2 and is unresponsive to arginine. Additionally, when arginine is depleted, the absence of CASTOR2 has no impact on mTORC1 activity.^[^ ^83^, ^84^ ^]^ This suggests that CASTOR2 does not act as an arginine sensor and may instead be influenced by alternative signals, such as cellular stress or yet unidentified factors.^[^ ^83^, ^84^ ^]^ SLC38A9 specifically senses arginine in the lysosome lumen. Arginine binds to the cytosol‐facing N‐terminal domain of SLC38A9, which then promotes its interaction with the Ragulator Rag GTPases. This interaction either switches or stabilizes RagA/B in the active state that binds to mTORC1, leading to the activation of mTORC1.^[^ ^62^, ^85^, ^86^, ^87^, ^88^ ^]^ Furthermore, transmembrane 4L six family member 5 (TM4SF5) has been suggested as an arginine sensor by interacting arginine via its extracellular loop. Upon binding to arginine, TM4SF5 moves from the plasma membrane to the lysosome, where it directly binds to and activates mTORC1.^[^ ^89^ ^]^

##### Methionine and One Carbon Sensor of mTORC1 Signaling

Unlike leucine and arginine, mTORC1 senses methionine via its intermediates, the methyl donor S‐adenosylmethionine (SAM), via two adenosylmethionine sensors, SAMTOR and PRMT1.^[^ ^90^, ^91^, ^92^ ^]^ These two sensors constitute the methionine‐sensing apparatus by influencing the activity of the GATOR1 complex. In the presence of methionine, SAM binds to SAMTOR, causing it to dissociate from GATOR1. This dissociation allows SAM‐bound PRMT1 to methylate NPRL2, thereby inhibiting the activity of GATOR1.^[^ ^90^, ^92^ ^]^ Furthermore, Unmet is suggested to act as a sensor for S‐adenosylmethionine (SAM) in Drosophila by binding to the GATOR2 complex.^[^ ^93^ ^]^ In yeast, methyltransferase Ppm1p sensed methionine via dictating the methylation status of the PP2A family of phosphatases. Methylated PP2A promotes dephosphorylation of Npr2p (analogous to NPRL2 in mammals) to reciprocally regulate TOR activation, cell growth and autophagy.^[^ ^94^ ^]^

##### Threonine Sensor of mTORC1 Signaling

Mitochondrial threonyl‐tRNA synthetase 2 (TARS2) has been suggested to act as a threonine sensor for mTORC1 signaling. In the presence of threonine, threonine bound TARS2 interacts with inactive Rags and enhancing the GTP loading of RagA, which in turns leading to the activation of mTORC1.^[^ ^95^ ^]^

In summary, it is important to note that while many specific amino acid sensors for mTORC1 signaling have been identified, the precise mechanisms of how these amino acids are sensed remain unclear. Insights are expected from the co‐structures of GATOR2 or GATOR1 with their respective sensors. Recently, two independent in vivo studies revealed the physiological role of Sestrin2 as a leucine sensor regulating mTORC1 in mice and flies. Further exploration of the in vivo functions of the other sensors is necessary.

## 4 mTORC1 and GCN2 Independent Amino Acids Sensing Pathway

Apart from the mTORC1 and GCN2‐dependent mechanisms, recent fascinating studies have uncovered that specific amino acids like valine, arginine, asparagine and aspartate could be recognized by unique sensors that influence DNA damage, metabolic reprogramming, and T cell activation. Here, we explore the sensing mechanisms of these three distinct amino acids and their functions.

### 4.1 HDAC6‐Mediated Valine Sensing

**Figure 4:**
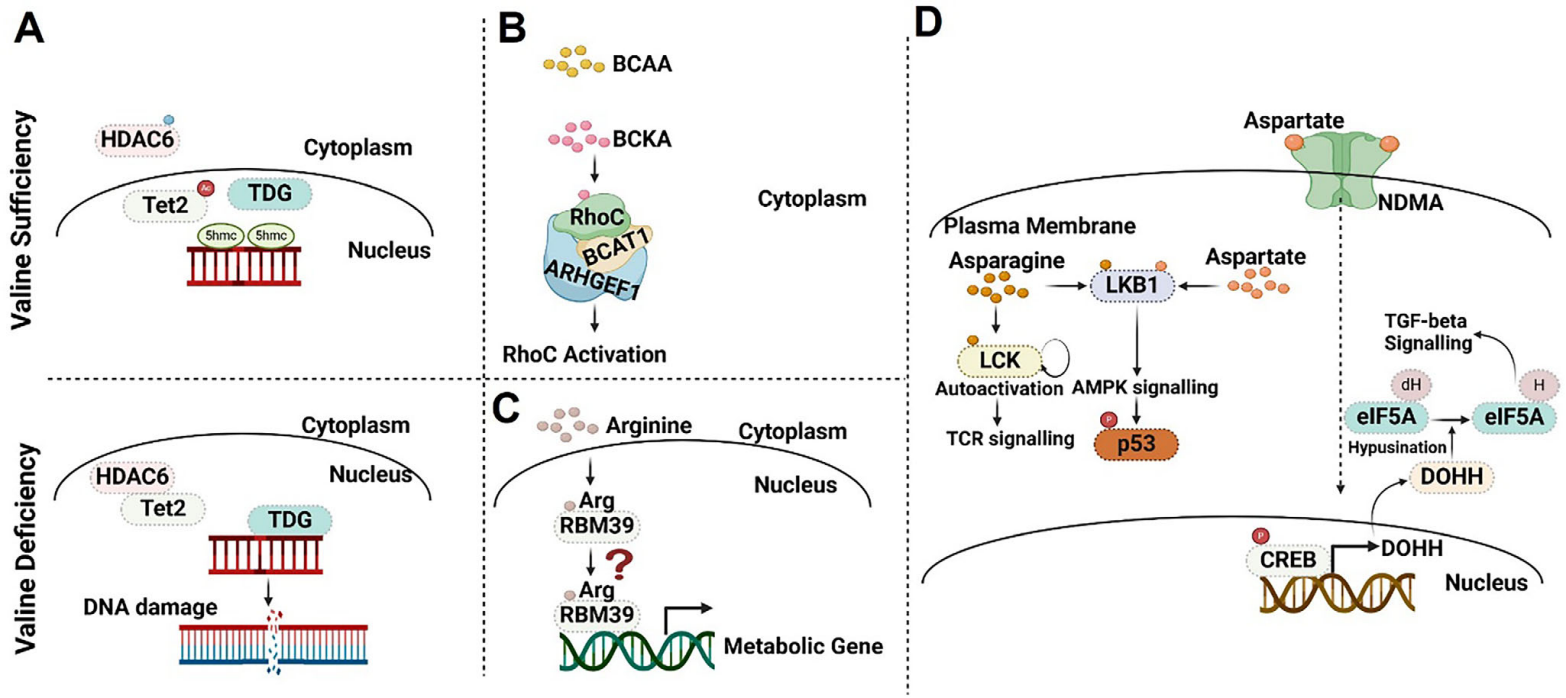
GCN2 and mTORC1 independent amino acid sensing. A) HDAC6‐mediated Valine sensing. B) RhoC‐mediated branch‐chain amino acid sensing. C) RBM39‐mediated arginine sensing. D) LCK and LKB1‐mediated asparagine sensing and NDMA and LKB1‐mediated aspartate sensing.

Valine, an essential branched‐chain amino acid (BCAA), is crucial for protein synthesis, energy production, and glucose metabolism. It also plays significant roles in both health and disease, including supporting mental function, enhancing the immune system, and contributing to the onset of leukemia. However, compared to another branched‐chain amino acid, leucine, the sensing mechanism of valine remains largely unexplored. Jin and her colleagues have revealed that human histone deacetylase 6 (HDAC6) acts as a direct sensor for valine^[^ ^96^ ^]^ (**Figure** **4**A). When valine levels are sufficient, it interacts with the SE14 repeat domain of HDAC6, blocking HDAC6's interaction with importin‐α1 and consequently retaining HDAC6 in the cytoplasm. In contrast, when valine is deficient, it fails to prevent HDAC6 from translocating to the nucleus. Once in the nucleus, HDAC6 deacetylates and activates TET2, initiating TET2‐TDG mediated DNA damage that inhibits cell proliferation.

Cells have developed diverse adaptive mechanism to maintain cellular hemostasis under nutrient stress condition. However, there are accumulating evidence also indicates that excessive nutrient stress can change cell fate and inhibits cell proliferation. Considering valine serves as an essential amino acid crucial for cellular function, its deficiency is perceived as a potential threat to cells, necessitating the establishment of a surveillance mechanism. This study proposes a model wherein cells detect the stress of valine deficiency through the HDAC6‐TET2 axis, which functions a nutrient check point to dictate cell fate via transmitting the valine availability to DNA demethylation and cell death. Furthermore, dietary restriction has been suggested as a potential adjunct therapy for cancer patients. This study demonstrated that restricting valine intake could increase DNA damage and enhance sensitivity to PARP inhibitors, offering significant translational implications. Given the proposed mechanism, disrupting the HDAC6‐mediated valine sensing pathway could present a new therapeutic option for treating cancer.

### 4.2 RhoC‐Mediated Branched‐Chain Amino Acid Sensing via Branched‐Chain α‐Keto Acid

Apart from the specific sensing mechanisms tailored to each branched‐chain amino acids (BCAAs), there are also general mechanisms that sense these amino acids through their shared intermediates, known as branched‐chain α‐keto acid (BCKA) (Figure 4B). BCKAs directly interact with the small GTPase protein RhoC, which leads to the assembly of a BCAT1‐BCKA‐RhoC‐ARHGEF1 membraneless metabolite‐protein complex, triggering the activation of RhoC.^[^ ^97^ ^]^ This branched‐chain amino acid sensing pathway effectively integrates BCAA metabolism with the regulation of RhoC, precisely modulating its transition between active and inactive states and is essential for promoting cell migration and tumorigenesis.

### 4.3 RBM39‐Mediated Arginine Sensing

The research group led by Michael N. Hall revealed that arginine accumulation in hepatocellular carcinoma (HCC) is due to increased uptake and reduced conversion of arginine to polyamines. This increase in arginine levels leads to its binding to the N‐terminal region of RBM39, triggering the transcriptional activation of metabolic genes involved in glucose, pyruvate, amino acids, NAD^+^, nucleotides, fatty acids, and aldehyde metabolism^[^ ^98^ ^]^ (Figure 4C). Moreover, RBM39 induces an increase in ASAN expression, which enhances arginine uptake, creating a positive feedback loop that sustains elevated arginine levels and supports oncogenic metabolism. Further research is needed to determine the dissociation constant (*K*_d_) for the arginine‐RBM39 interaction, assess whether it aligns with arginine fluctuations during hepatocellular carcinoma (HCC) progression, and elucidate how arginine‐bound RBM39 influences the transcription of downstream metabolic genes. Additionally, it would be interesting to explore the potential interplay between CASTOR1 and RBM39 in arginine sensing. Do they exhibit distinct sensitivities, tissue‐specific distributions, or function independently or cooperatively? These questions remain to be investigated.

Tumor cells that lack urea cycle enzymes are unable to synthesize arginine, necessitating the uptake of external arginine to survive—a condition termed arginine auxotrophy. This reliance has prompted the development of therapeutic strategies using circulating enzymes that degrade arginine to target and kill arginine‐auxotrophic cancer cells. However, clinical improvements have been minimal. This intriguing study revealed first time that targeting the cancer‐specific arginine binding protein RBM39 with indisulam to promote its degradation could treat hepatocellular carcinoma (HCC) with high tumoral arginine levels across various models. This approach offers a novel therapeutic strategy for treating arginine‐auxotrophic cancer cells, holding significant translational potential.

### 4.4 LCK and LKB1‐Mediated Asparagine Sensing

Nutrition, including glucose, amino acids, and lipids, is identified as signal four essential for mediating T cell immunity. LCK is believed to bind asparagine, facilitating the activation and effector functions of CD8^+^ T cells against tumor cells.^[^ ^99^ ^]^ Mechanistically, the binding of asparagine to the kinase domain of LCK leads to enhanced phosphorylation at Tyr 394 and Tyr 505, increasing LCK activity and amplifying T‐cell‐receptor signaling (Figure 4D).^[^ ^99^ ^]^ Limiting asparagine uptake, neither through an asparagine‐restricted diet, administering ASNase, or inhibiting the asparagine transporter SLC1A5, impairs the response of CD8^+^ T cells and their anti‐tumor effects.^[^ ^99^ ^]^ Additional research is required to determine the precise intracellular asparagine concentration in CD8^+^ T cells and to assess if variations in asparagine levels during cancer progression can sufficiently activate anti‐tumor effects through LCK interactions. In addition, the same research group also reported that asparagine and aspartate can directly binds to LKB1 with a *K*_d_ around 45.4 × 10^−6^ and 64.6 × 10^−6^ m, respectively, levels that coincide with asparagine fluctuations in tumors with p53 mutations, which exhibits elevated asparagine due to altered ASAN expression.^[^ ^100^ ^]^ This study indicates that LKB1 functions as a natural sensor for Asp‐Asn homeostasis^[^ ^100^ ^]^ (Figure 4D).

An independent study has shown that asparagine plays a role in promoting breast tumor‐cell metastasis, and reducing its availability can decrease metastasis without impacting the primary tumor's growth. Therefore, the influence of asparagine on tumor progression may depend on the context and timing; particularly, in later stages of metastasis when tumor cells have bypassed the immune surveillance system, asparagine can enhance tumor cell migration and metastasis.^[^ ^101^ ^]^ Consequently, targeting asparagine might offer varying therapeutic benefits in cancer treatment, necessitating further clinical exploration.

### 4.5 NDMA‐Mediated Aspartate Sensing

Aspartate and asparagine, while structurally similar and derived from the same precursor, aspartic acid, exhibit distinct biological roles, underscoring their unique roles in the body's systems.

In a model of breast cancer metastasis to the lung, aspartate levels are found to be elevated in the lung interstitial fluid. Mechanistically, aspartate binds to the N‐methyl‐d‐aspartate (NMDA) type subunit 2D (GRIN2D), enhancing the CREB‐dependent expression of deoxyhypusine hydroxylase (DOHH), which is crucial for hypusination of the non‐classical translation initiation factor eIF5A^[^ ^102^ ^]^ (Figure 4D). This, in turn, supports a translational program centered around TGFβ signaling, which promotes collagen production in lung‐disseminated breast cancer cells. This study highlights aspartate's multifaceted role, extending beyond its traditional involvement in synthesizing proteins and nucleotides and managing redox homeostasis. Aspartate also acts as a signaling molecule that regulates the translation process to increase collagen synthesis, indicating its potential to prepare the pre‐metastatic niche for aggressive metastasis.

### 4.6 Perspective

In recent years, our understanding of the molecular basis of amino acid sensing and its respective functions has broadened. The sensors of amino acids vary widely in structure and function, ranging from membrane‐anchored transceptors such as Tsr and Tar in prokaryotes and Ssy in yeast, to cytosolic sensing pathways like GCN2 and mTORC1, along with other recently identified mechanisms independent of GCN2 and mTORC1. There are direct sensing mechanisms that interact directly with amino acids, as well as indirect sensing mechanisms that operate through intermediates of amino acids, such as methionine sensing via S‐adenosylmethionine (SAM), or nitrogen deficiency sensing via alpha‐ketoglutarate (α‐KG) in yeast and BCAA sensing via branched‐chain α‐keto acid. Cellular and organismal functions range from maintaining intrinsic fitness to orchestrating nutrient stress responses and mediating cancer metabolism. Despite advancements in this field, many questions about amino acid sensing remain.

### 4.7 How to Define and Identify Direct Amino Acid Sensor?

Among the 20 standard amino acids, only the sensing mechanisms for leucine, arginine, valine, and asparagine have been identified to date. It remains uncertain whether sensors for the other amino acids exist and how they might function. In the context of mTORC1‐dependent amino acid sensing, it is not yet clear if this pathway can sense all amino acids. Moreover, RNA and DNA aptamers have been identified that bind to leucine and glutamine, suggesting that nucleic acids could potentially act as amino acid sensors. This raises the question of the nature of these specific sensors and their roles within amino acid sensing pathways. Additionally, there are technical challenges in identifying amino acid sensors. Traditionally, methods such as conjugating biotin to specific amino acid groups or using click chemistry to probe amino acids, followed by affinity purification and mass spectrometry, have been employed. However, these methods often face challenges with low‐affinity interactions that are hard to detect systematically. Additionally, attaching biotin or using click chemistry on amino acids may impair their function, complicating the identification of authentic sensors through mass spectrometry. Alternative approaches like the MIDAS method developed by Dr. Jared Rutter's group,^[^ ^103^ ^]^ and cellular thermal shift assay (CETSA),^[^ ^104^ ^]^ thermal proteome profiling (TPP),^[^ ^105^ ^]^ peptide‐centric approaches including limited proteolysis–mass spectrometry (LiP–MS),^[^ ^106^, ^107^ ^]^ LiP‐Quant (an updated version of LiP‐MS)^[^ ^108^ ^]^ and peptide‐centric local stability assay (PELS)^[^ ^109^ ^]^ are promising for systematically identifying amino acid‐binding proteins. Beyond biochemical methods, genetic approaches such as genome‐wide CRISPR screens could provide critical insights into novel amino acid sensing pathways.

### 4.8 What Are the Physiological and Pathological Roles of Distinct Amino Acid Sensing Pathways?

To date, Sestrin2 has been the only identified physiological sensor of leucine at the organismal level. Deleting Sestrin1 and Sestrin2 disrupts mTORC1 inhibition under leucine‐deficient conditions, leading to a rapid loss of white adipose and muscle tissue essential for maintaining organismal homeostasis.^[^ ^77^ ^]^ Further studies in flies have demonstrated that Sestrin's ability to sense leucine not only activates mTORC1 in glial cells but also guides dietary choices, assisting them in selecting between leucine‐rich and leucine‐poor foods, crucial for neural metabolic regulation.^[^ ^110^ ^]^ Additionally, PRMT1‐mediated methionine sensing plays a vital role in alleviating insulin resistance induced by methionine restriction in aged mice.^[^ ^90^ ^]^ Similarly, HDAC6‐mediated valine sensing is pivotal for inducing tumor regression under valine restriction by determining cell fate in response to nutrient stress, thus highlighting the pathological functions of these specific amino acid sensing pathways.^[^ ^96^ ^]^ The full range of physiological and pathological roles of amino acid sensing pathways, beyond maintaining homeostasis or determining cell fate, remains unclear.

### 4.9 The Organ and Organelle Specific Amino Acid Sensing Pathway

The levels and locations of amino acids vary among various organs and organelles, facilitating amino acid compartmentalization. Notably, in the mTORC1 signaling pathway, the leucine sensors Sestrin2 and SAR1B differ in both their distribution across tissues and their sensitivity to leucine, each recognizing unique structural aspects of leucine.^[^ ^75^, ^76^ ^]^ Sestrins predominantly occur in periportal and midlobular hepatocytes, strategically positioned nearer to nutrient sources, thereby ensuring better access to leucine and illustrating the localized leucine detection capabilities of mTORC1.^[^ ^77^ ^]^ Predominantly situated in lysosomes, mTORC1 integrates amino acids from both the cytosol and lysosomes.^[^ ^53^ ^]^ This leads to an intriguing question: Could there be specific amino acid sensing mechanisms tailored to individual organs or organelles that play a role in their unique functions? Further research is needed to explore this possibility.

### 4.10 Amino Acid Sensors across Evolution

Different species have unique needs for amino acids, accompanied by distinct mechanisms to detect these molecules—a concept that's gaining support through recent studies. The role of these unique sensing pathways in the evolutionary journey from prokaryotes to eukaryotes, and from simple yeasts to complex mammals, remains a mystery. Throughout evolution, various cells, tissues, and organisms have adapted to unique nutritional environments to ensure survival and reproduction. To thrive in these diverse niches, cells have developed specialized sensors that respond to the specific biochemical and biophysical characteristics of their environments. As a result, it is likely that various sensors have developed across different metazoan phyla. Indeed, recent findings emphasize the wide range of amino acid sensing mechanisms that have emerged across species, underscoring their evolutionary diversity. For example, the arginine sensors of mTORC1, CASTOR1, and SLC38A9 exist in mice and humans, but are not found in simpler organisms such as *D. melanogaster* or *C. elegans*.^[^ ^84^, ^85^ ^]^ Conversely, the leucine sensors Sestrins and SAR1B are conserved from *C. elegans* to mammals.^[^ ^75^, ^76^, ^77^, ^110^ ^]^ Additionally, LRS is conserved across a range from yeast to mammals.^[^ ^78^ ^]^ Similarly, variations in methionine sensors have been noted in mammals, flies and yeast. The valine sensing mechanism facilitated by HDAC6 that is unique to primates, posing an interesting question about primates' heightened sensitivity to valine depletion.^[^ ^96^ ^]^ This sensitivity is underscored by findings that human cells are more vulnerable than mouse cells under certain stress conditions, such as increased susceptibility to oxidative damage in human astrocytes' mitochondria compared to those in mice. Additionally, both HDAC6 and valine are implicated in neuronal function regulation, suggesting that this primate‐specific valine sensing mechanism might contribute to distinguishing primate neurological features from those of other mammals. The function relevance of these diverse sensing mechanism across evolution remains to further explored.

#### 4.10.1 How to Define the Physiological Concentration of Amino Acids?

A crucial aspect of amino acid sensors is that their dissociation constant—between amino acids and proteins—should align with the physiological concentration fluctuations, which can trigger downstream effects. This alignment is a key criterion for defining a true physiological amino acid sensor. Beyond their basic physiological roles, amino acid sensing pathways are also critical in adapting to various nutrient stresses, requiring that the dissociation constant remains within the appropriate range under different stress conditions. Additionally, the analysis of amino acids in whole cells, tissues, or organisms alone is insufficient to confirm the presence of a genuine amino acid sensor. There is a pressing need for new methodologies to study organelle metabolism. Techniques such as OrganelleIP^[^ ^111^, ^112^, ^113^, ^114^, ^115^ ^]^ or the development of genetically encoded biosensors,^[^ ^116^ ^]^ which can measure local amino acid concentrations specifically in different organs or organelles, are essential for advancing our understanding in this field.

#### 4.10.2 How to Target Amino Acid Sensor?

To date, the arginine binding protein RBM39 and the leucine sensor Sestrin2 have been suggested as targets for indisulam and NV‐5138 for treating cancer and depression,^[^ ^98^, ^117^ ^]^ respectively. Furthermore, studies demonstrate that NV‐5138, by acting on Sestrin2, can effectively prevent or restore diminished gyrification and sulcation in lissencephaly spectrum disorders.^[^ ^118^ ^]^ Additionally, GSK3368715 has been suggested to target PRMT1, which could disrupt methionine sensing in mTORC1 and potentially alleviate insulin resistance in aged mice.^[^ ^90^ ^]^ This leads to an important question: Are amino acid sensors promising drug targets? If so, how can various amino acid sensors be targeted to treat different diseases?

## Conflict of Interest

The authors declare no conflict of interest.

## References

[1] Z. N. Ling , Y. F. Jiang , J. N. Ru , J. H. Lu , B. Ding , J. Wu , Signal Transduction Targeted Ther. 2023, 8, 345. 10.1038/s41392-023-01569-3 PMC10497558 37699892

[2] X. Hu , F. Guo , Endocr. Rev. 2021, 42, 56. 33053153 10.1210/endrev/bnaa026

[3] G. Wu , Amino Acids 2009, 37, 1. 19301095 10.1007/s00726-009-0269-0

[4] P. J. Reeds , J. Nutr. 2000, 130, 1835S. 10867060 10.1093/jn/130.7.1835S

[5] G. D'Andrea , Biochem. Educ. 2000, 28, 27. 10717451 10.1016/s0307-4412(98)00271-4

[6] R. Colin , V. Sourjik , Curr. Opin. Microbiol. 2017, 39, 24. 28822274 10.1016/j.mib.2017.07.004

[7] E. Krasnopeeva , U. E. Barboza‐Perez , J. Rosko , T. Pilizota , C. J. Lo , Methods 2021, 193, 5. 32640316 10.1016/j.ymeth.2020.06.012

[8] H. Szurmant , G. W. Ordal , Microbiol. Mol. Biol. Rev. 2004, 68, 301. 15187186 10.1128/MMBR.68.2.301-319.2004 PMC419924

[9] R. Mesibov , G. W. Ordal , J. Adler , J. Gen. Physiol. 1973, 62, 203. 4578974 10.1085/jgp.62.2.203 PMC2226111

[10] V. Sourjik , H. C. Berg , Nature 2004, 428, 437. 15042093 10.1038/nature02406

[11] E. A. Wang , D. E. Koshland Jr , Proc. Natl. Acad. Sci. USA 1980, 77, 7157. 6784119 10.1073/pnas.77.12.7157 PMC350460

[12] M. Welch , K. Oosawa , S. Aizawa , M. Eisenbach , Proc. Natl. Acad. Sci. USA 1993, 90, 8787. 8415608 10.1073/pnas.90.19.8787 PMC47445

[13] Y. Chang , K. Zhang , B. L. Carroll , X. Zhao , N. W. Charon , S. J. Norris , M. A. Motaleb , C. Li , J. Liu , Nat. Struct. Mol. Biol. 2020, 27, 1041. 32895555 10.1038/s41594-020-0497-2 PMC8129871

[14] J. F. Hess , K. Oosawa , N. Kaplan , M. I. Simon , Cell 1988, 53, 79. 3280143 10.1016/0092-8674(88)90489-8

[15] J. F. Hess , K. Oosawa , P. Matsumura , M. I. Simon , Proc. Natl. Acad. Sci. USA 1987, 84, 7609. 3313398 10.1073/pnas.84.21.7609 PMC299349

[16] H. Kondoh , C. B. Ball , J. Adler , Proc. Natl. Acad. Sci. USA 1979, 76, 260. 370826 10.1073/pnas.76.1.260 PMC382918

[17] K. A. Borkovich , N. Kaplan , J. F. Hess , M. I. Simon , Proc. Natl. Acad. Sci. USA 1989, 86, 1208. 2645576 10.1073/pnas.86.4.1208 PMC286655

[18] R. M. Weis , D. E. Koshland Jr , Proc. Natl. Acad. Sci. USA 1988, 85, 83. 2829179 10.1073/pnas.85.1.83 PMC279486

[19] G. H. Wadhams , J. P. Armitage , Nat. Rev. Mol. Cell Biol. 2004, 5, 1024. 15573139 10.1038/nrm1524

[20] J. A. Leigh , J. A. Dodsworth , Annu. Rev. Microbiol. 2007, 61, 349. 17506680 10.1146/annurev.micro.61.080706.093409

[21] K. Forchhammer , Front. Biosci. 2007, 12, 358. 17127304 10.2741/2069

[22] E. R. Stadtman , J. Biol. Chem. 2001, 276, 44357. 11585846 10.1074/jbc.R100055200

[23] M. S. Brown , A. Segal , E. R. Stadtman , Proc. Natl. Acad. Sci. USA 1971, 68, 2949. 4399832 10.1073/pnas.68.12.2949 PMC389567

[24] J. H. Mangum , G. Magni , E. R. Stadtman , Arch. Biochem. Biophys. 1973, 158, 514. 4150122 10.1016/0003-9861(73)90543-2

[25] S. P. Adler , D. Purich , E. R. Stadtman , J. Biol. Chem. 1975, 250, 6264. 239942

[26] P. Jiang , J. A. Peliska , A. J. Ninfa , Biochemistry 1998, 37, 12802. 9737857 10.1021/bi980666u

[27] P. Jiang , J. A. Peliska , A. J. Ninfa , Biochemistry 1998, 37, 12795. 9737856 10.1021/bi9802420

[28] W. C. van Heeswijk , S. Hoving , D. Molenaar , B. Stegeman , D. Kahn , H. V. Westerhoff , Mol. Microbiol. 1996, 21, 133. 8843440 10.1046/j.1365-2958.1996.6281349.x

[29] G. Coutts , G. Thomas , D. Blakey , M. Merrick , EMBO J. 2002, 21, 536. 11847102 10.1093/emboj/21.4.536 PMC125854

[30] M. M. Davis , F. J. Alvarez , K. Ryman , A. A. Holm , P. O. Ljungdahl , Y. Engstrom , PLoS One 2011, 6, 27434. 10.1371/journal.pone.0027434 PMC3215725 22110651

[31] H. Klasson , G. R. Fink , P. O. Ljungdahl , Mol. Cell. Biol. 1999, 19, 5405. 10409731 10.1128/mcb.19.8.5405 PMC84383

[32] T. Didion , B. Regenberg , M. U. Jorgensen , M. C. Kielland‐Brandt , H. A. Andersen , Mol. Microbiol. 1998, 27, 643. 9489675 10.1046/j.1365-2958.1998.00714.x

[33] M. Conrad , J. Schothorst , H. N. Kankipati , G. Van Zeebroeck , M. Rubio‐Texeira , J. M. Thevelein , FEMS Microbiol. Rev. 2014, 38, 254. 24483210 10.1111/1574-6976.12065 PMC4238866

[34] F. Bernard , B. Andre , Mol. Microbiol. 2001, 41, 489. 11489133 10.1046/j.1365-2958.2001.02538.x

[35] I. Iraqui , S. Vissers , F. Bernard , J. O. de Craene , E. Boles , A. Urrestarazu , B. Andre , Mol. Cell. Biol. 1999, 19, 989. 9891035 10.1128/mcb.19.2.989 PMC116030

[36] T. Pfirrmann , S. Heessen , D. J. Omnus , C. Andreasson , P. O. Ljungdahl , Mol. Cell. Biol. 2010, 30, 3299. 20421414 10.1128/MCB.00323-10 PMC2897576

[37] D. J. Omnus , T. Pfirrmann , C. Andreasson , P. O. Ljungdahl , Mol. Biol. Cell 2011, 22, 2754. 21653827 10.1091/mbc.E11-04-0282 PMC3145550

[38] F. Abdel‐Sater , C. Jean , A. Merhi , S. Vissers , B. Andre , J. Biol. Chem. 2011, 286, 12006. 21310956 10.1074/jbc.M110.200592 PMC3069403

[39] P. Poulsen , R. F. Gaber , M. C. Kielland‐Brandt , Mol. Membr. Biol. 2008, 25, 164. 18307103 10.1080/09687680701771917

[40] A. Efeyan , W. C. Comb , D. M. Sabatini , Nature 2015, 517, 302. 25592535 10.1038/nature14190 PMC4313349

[41] R. Sood , A. C. Porter , D. A. Olsen , D. R. Cavener , R. C. Wek , Genetics 2000, 154, 787. 10655230 10.1093/genetics/154.2.787 PMC1460965

[42] J. J. Berlanga , J. Santoyo , C. De Haro , Eur. J. Biochem. 1999, 265, 754. 10504407 10.1046/j.1432-1327.1999.00780.x

[43] A. G. Hinnebusch , Proc. Natl. Acad. Sci. USA 1984, 81, 6442.6387704

[44] D. Krokowski , J. Han , M. Saikia , M. Majumder , C L. Yuan , B.‐J. Guan , E. Bevilacqua , O. Bussolati , S. Bröer , P. Arvan , M. Tchórzewski , M D. Snider , M. Puchowicz , C M. Croniger , S R. Kimball , T. Pan , A E. Koromilas , R J. Kaufman , M. Hatzoglou , J. Biol. Chem. 2013, 288, 17202. 23645676 10.1074/jbc.M113.466920 PMC3682525

[45] H P. Harding , Y. Zhang , H. Zeng , I. Novoa , P D. Lu , M. Calfon , N. Sadri , C. Yun , B. Popko , R. Paules , D F. Stojdl , J C. Bell , T. Hettmann , J M. Leiden , D. Ron , Mol. Cell 2003, 11, 619. 12667446 10.1016/s1097-2765(03)00105-9

[46] H. P. Harding , I. Novoa , Y. Zhang , H. Zeng , R. Wek , M. Schapira , D. Ron , Mol. Cell 2000, 6, 1099. 11106749 10.1016/s1097-2765(00)00108-8

[47] P. Bunpo , A. Dudley , J. K. Cundiff , D. R. Cavener , R. C. Wek , T. G. Anthony , J. Biol. Chem. 2009, 284, 32742. 19783659 10.1074/jbc.M109.047910 PMC2781691

[48] W. B'Chir , A. C. Maurin , V. Carraro , J. Averous , C. Jousse , Y. Muranishi , L. Parry , G. Stepien , P. Fafournoux , A. Bruhat , Nucleic Acids Res. 2013, 41, 7683. 23804767 10.1093/nar/gkt563 PMC3763548

[49] H. O. Jin , S. E. Hong , J. Y. Kim , S. K. Jang , I. C. Park , Cell Death Dis. 2021, 12, 1127. 34862383 10.1038/s41419-021-04417-w PMC8642548

[50] H. Ming , J. Tan , S. Y. Cao , C. P. Yu , Y. T. Qi , C. Wang , L. Zhang , Y. Liu , J. Yuan , M. Yin , Q. Y. Lei , Nat. Metab. 2025, 7, 120.. 39753713 10.1038/s42255-024-01179-5

[51] V. Albert , M. N. Hall , Curr. Opin. Cell Biol. 2015, 33, 55. 25554914 10.1016/j.ceb.2014.12.001

[52] J. Kim , K. L. Guan , Nat. Cell Biol. 2019, 21, 63. 30602761 10.1038/s41556-018-0205-1

[53] G. Y. Liu , D. M. Sabatini , Nat. Rev. Mol. Cell Biol. 2020, 21, 183. 31937935 10.1038/s41580-019-0199-y PMC7102936

[54] C. Goul , R. Peruzzo , R. Zoncu , Nat. Rev. Mol. Cell Biol. 2023, 24, 857. 37612414 10.1038/s41580-023-00641-8

[55] X. Long , Y. Lin , S. Ortiz‐Vega , K. Yonezawa , J. Avruch , Curr. Biol. 2005, 15, 702. 15854902 10.1016/j.cub.2005.02.053

[56] K. Inoki , Y. Li , T. Xu , K. L. Guan , Genes Dev. 2003, 17, 1829. 12869586 10.1101/gad.1110003 PMC196227

[57] E. Kim , P. Goraksha‐Hicks , L. Li , T. P. Neufeld , K. L. Guan , Nat. Cell Biol. 2008, 10, 935. 18604198 10.1038/ncb1753 PMC2711503

[58] Y. Sancak , T. R. Peterson , Y. D. Shaul , R. A. Lindquist , C. C. Thoreen , L. Bar‐Peled , D. M. Sabatini , Science 2008, 320, 1496. 18497260 10.1126/science.1157535 PMC2475333

[59] S. Menon , C. C. Dibble , G. Talbott , G. Hoxhaj , A. J. Valvezan , H. Takahashi , L. C. Cantley , B. D. Manning , Cell 2014, 156, 771. 24529379 10.1016/j.cell.2013.11.049 PMC4030681

[60] J. L. Jewell , Y. C. Kim , R. C. Russell , F. X. Yu , H. W. Park , S. W. Plouffe , V. S. Tagliabracci , K. L. Guan , Science 2015, 347, 194. 25567907 10.1126/science.1259472 PMC4384888

[61] J. D. Thomas , Y. J. Zhang , Y. H. Wei , J. H. Cho , L. E. Morris , H. Y. Wang , X. F. Zheng , Cancer Cell 2014, 26, 754. 25446900 10.1016/j.ccell.2014.09.008 PMC4288827

[62] K. Shen , D. M. Sabatini , Proc. Natl. Acad. Sci. USA 2018, 115, 9545. 30181260 10.1073/pnas.1811727115 PMC6156610

[63] L. Bar‐Peled , L. D. Schweitzer , R. Zoncu , D. M. Sabatini , Cell 2012, 150, 1196. 22980980 10.1016/j.cell.2012.07.032 PMC3517996

[64] C. Y. Chung , H. R. Shin , C. A. Berdan , B. Ford , C. C. Ward , J. A. Olzmann , R. Zoncu , D. K. Nomura , Nat. Chem. Biol. 2019, 15, 776. 31285595 10.1038/s41589-019-0308-4 PMC6641988

[65] R. Zoncu , L. Bar‐Peled , A. Efeyan , S. Wang , Y. Sancak , D. M. Sabatini , Science 2011, 334, 678. 22053050 10.1126/science.1207056 PMC3211112

[66] Y. Sancak , L. Bar‐Peled , R. Zoncu , A. L. Markhard , S. Nada , D. M. Sabatini , Cell 2010, 141, 290. 20381137 10.1016/j.cell.2010.02.024 PMC3024592

[67] Z. Y. Tsun , L. Bar‐Peled , L. Chantranupong , R. Zoncu , T. Wang , C. Kim , E. Spooner , D. M. Sabatini , Mol. Cell 2013, 52, 495. 24095279 10.1016/j.molcel.2013.09.016 PMC3867817

[68] L. Bar‐Peled , L. Chantranupong , A. D. Cherniack , W. W. Chen , K. A. Ottina , B. C. Grabiner , E. D. Spear , S. L. Carter , M. Meyerson , D. M. Sabatini , Science 2013, 340, 1100. 23723238 10.1126/science.1232044 PMC3728654

[69] M. Peng , N. Yin , M. O. Li , Nature 2017, 543, 433. 28199315 10.1038/nature21378 PMC5570594

[70] R L. Wolfson , L. Chantranupong , G A. Wyant , X. Gu , J M. Orozco , K. Shen , K J. Condon , S. Petri , J. Kedir , S M. Scaria , M. Abu‐Remaileh , W N. Frankel , D M. Sabatini , Nature 2017, 543, 438. 28199306 10.1038/nature21423 PMC5360989

[71] G. Yan , J. Yang , W. Li , A. Guo , J. Guan , Y. Liu , Nat. Cell Biol. 2023, 25, 754. 37037994 10.1038/s41556-023-01123-x

[72] C. Jiang , X. Dai , S. He , H. Zhou , L. Fang , J. Guo , S. Liu , T. Zhang , W. Pan , H. Yu , T. Fu , D. Li , H. Inuzuka , P. Wang , J. Xiao , W. Wei , Mol. Cell 2023, 83, 74. 36528027 10.1016/j.molcel.2022.11.021 PMC11027793

[73] M. L. Valenstein , K. B. Rogala , P. V. Lalgudi , E. J. Brignole , X. Gu , R. A. Saxton , L. Chantranupong , J. Kolibius , J. P. Quast , D. M. Sabatini , Nature 2022, 607, 610. 35831510 10.1038/s41586-022-04939-z PMC9464592

[74] R. A. Saxton , K. E. Knockenhauer , R. L. Wolfson , L. Chantranupong , M. E. Pacold , T. Wang , T. U. Schwartz , D. M. Sabatini , Science 2016, 351, 53. 26586190 10.1126/science.aad2087 PMC4698039

[75] R. L. Wolfson , L. Chantranupong , R. A. Saxton , K. Shen , S. M. Scaria , J. R. Cantor , D. M. Sabatini , Science 2016, 351, 43. 26449471 10.1126/science.aab2674 PMC4698017

[76] J. Chen , Y. Ou , R. Luo , J. Wang , D. Wang , J. Guan , Y. Li , P. Xia , P. R. Chen , Y. Liu , Nature 2021, 596, 281. 34290409 10.1038/s41586-021-03768-w

[77] A L. Cangelosi , A M. Puszynska , J M. Roberts , A. Armani , T P. Nguyen , J B. Spinelli , T. Kunchok , B. Wang , S. H. Chan , C A. Lewis , W C. Comb , G W. Bell , A. Helman , D M. Sabatini , Science 2022, 377, 47. 35771919 10.1126/science.abi9547 PMC10049859

[78] J. M. Han , S. J. Jeong , M. C. Park , G. Kim , N. H. Kwon , H. K. Kim , S. H. Ha , S. H. Ryu , S. Kim , Cell 2012, 149, 410. 22424946 10.1016/j.cell.2012.02.044

[79] X.‐D. He , W. Gong , J.‐N. Zhang , J. Nie , C.‐F. Yao , F.‐S. Guo , Y. Lin , X.‐H. Wu , F. Li , J. Li , W.‐C. Sun , E.‐D. Wang , Y.‐P. An , H.‐R. Tang , G.‐Q. Yan , P.‐Y. Yang , Y. Wei , Y.‐Z. Mao , P.‐C. Lin , J.‐Y. Zhao , Y. Xu , W. Xu , S.‐M. Zhao , Cell Metab. 2018, 27, 151. 29198988 10.1016/j.cmet.2017.10.015

[80] R. L. Wolfson , D. M. Sabatini , Cell Metab. 2017, 26, 301. 28768171 10.1016/j.cmet.2017.07.001 PMC5560103

[81] S. Kim , I. Yoon , J. Son , J. Park , K. Kim , J. H. Lee , S. Y. Park , B. S. Kang , J. M. Han , K. Y. Hwang , S. Kim , Cell Rep. 2021, 35, 109031. 33910001 10.1016/j.celrep.2021.109031

[82] G. Bonfils , M. Jaquenoud , S. Bontron , C. Ostrowicz , C. Ungermann , C. De Virgilio , Mol. Cell 2012, 46, 105. 22424774 10.1016/j.molcel.2012.02.009

[83] R. A. Saxton , L. Chantranupong , K. E. Knockenhauer , T. U. Schwartz , D. M. Sabatini , Nature 2016, 536, 229. 27487210 10.1038/nature19079 PMC4988899

[84] L. Chantranupong , S. M. Scaria , R. A. Saxton , M. P. Gygi , K. Shen , G. A. Wyant , T. Wang , J. W. Harper , S. P. Gygi , D. M. Sabatini , Cell 2016, 165, 153. 26972053 10.1016/j.cell.2016.02.035 PMC4808398

[85] G. A. Wyant , M. Abu‐Remaileh , R. L. Wolfson , W. W. Chen , E. Freinkman , L. V. Danai , M. G. Vander Heiden , D. M. Sabatini , Cell 2017, 171, 642. 29053970 10.1016/j.cell.2017.09.046 PMC5704964

[86] M. Rebsamen , L. Pochini , T. Stasyk , M E. G. de Araújo , M. Galluccio , R K. Kandasamy , B. Snijder , A. Fauster , E L. Rudashevskaya , M. Bruckner , S. Scorzoni , P A. Filipek , K V. M. Huber , J W. Bigenzahn , L X. Heinz , C. Kraft , K L. Bennett , C. Indiveri , L A. Huber , G. Superti‐Furga , Nature 2015, 519, 477. 25561175 10.1038/nature14107 PMC4376665

[87] J. Jung , H. M. Genau , C. Behrends , Mol. Cell. Biol. 2015, 35, 2479. 25963655 10.1128/MCB.00125-15 PMC4475919

[88] S. A. Fromm , R. E. Lawrence , J. H. Hurley , Nat. Struct. Mol. Biol. 2020, 27, 1017. 32868926 10.1038/s41594-020-0490-9 PMC7644641

[89] J. W. Jung , S. J. Y. Macalino , M. Cui , J. E. Kim , H. J. Kim , D. G. Song , S. H. Nam , S. Kim , S. Choi , J. W. Lee , Cell Metab. 2019, 29, 1306. 30956113 10.1016/j.cmet.2019.03.005

[90] C. Jiang , J. Liu , S. He , W. Xu , R. Huang , W. Pan , X. Li , X. Dai , J. Guo , T. Zhang , H. Inuzuka , P. Wang , J M. Asara , J. Xiao , W. Wei , Cell Metab. 2023, 35, 2183. 38006878 10.1016/j.cmet.2023.11.001 PMC11192564

[91] X. Tang , Y. Zhang , G. Wang , C. Zhang , F. Wang , J. Shi , T. Zhang , J. Ding , Sci. Adv. 2022, 8, abn3868. 10.1126/sciadv.abn3868 PMC10883374 35776786

[92] X. Gu , J. M. Orozco , R. A. Saxton , K. J. Condon , G. Y. Liu , P. A. Krawczyk , S. M. Scaria , J. W. Harper , S. P. Gygi , D. M. Sabatini , Science 2017, 358, 813. 29123071 10.1126/science.aao3265 PMC5747364

[93] G. Y. Liu , P. Jouandin , R. E. Bahng , N. Perrimon , D. M. Sabatini , Nat. Commun. 2024, 15, 2517. 38514639 10.1038/s41467-024-46680-3 PMC10957897

[94] B. M. Sutter , X. Wu , S. Laxman , B. P. Tu , Cell 2013, 154, 403. 23870128 10.1016/j.cell.2013.06.041 PMC3774293

[95] S. H. Kim , J. H. Choi , P. Wang , C. D. Go , G. G. Hesketh , A. C. Gingras , S. M. Jafarnejad , N. Sonenberg , Mol. Cell 2021, 81, 398. 33340489 10.1016/j.molcel.2020.11.036

[96] J. Jin , T. Meng , Y. Yu , S. Wu , C.‐C. Jiao , S. Song , Y.‐X. Li , Y. Zhang , Y.‐Y. Zhao , X. Li , Z. Wang , Y.‐F. Liu , R. Huang , J. Qin , Y. Chen , H. Cao , X. Tan , X. Ge , C. Jiang , J. Xue , J. Yuan , D. Wu , W. Wu , C.‐Z. Jiang , P. Wang , Nature 2025, 637, 215. 39567688 10.1038/s41586-024-08248-5

[97] L. Qian , N. Li , X.‐C. Lu , M. Xu , Y. Liu , K. Li , Y. Zhang , K. Hu , Y.‐T. Qi , J. Yao , Y.‐L. Wu , W. Wen , S. Huang , Z.‐J. Chen , M. Yin , Q.‐Y. Lei , Nat. Metab. 2023, 5, 1159. 37337119 10.1038/s42255-023-00818-7

[98] D. Mossmann , C. Müller , S. Park , B. Ryback , M. Colombi , N. Ritter , D. Weißenberger , E. Dazert , M. Coto‐Llerena , S. Nuciforo , L. Blukacz , C. Ercan , V. Jimenez , S. Piscuoglio , F. Bosch , L M. Terracciano , U. Sauer , M H. Heim , M N. Hall , Cell 2023, 186, 5068. 37804830 10.1016/j.cell.2023.09.011 PMC10642370

[99] J. Wu , G. Li , L. Li , D. Li , Z. Dong , P. Jiang , Nat. Cell Biol. 2021, 23, 75. 33420490 10.1038/s41556-020-00615-4

[100] L. Deng , P. Yao , L. Li , F. Ji , S. Zhao , C. Xu , X. Lan , P. Jiang , Nat. Commun. 2020, 11, 1755. 32273511 10.1038/s41467-020-15573-6 PMC7145870

[101] S R. V. Knott , E. Wagenblast , S. Khan , S Y. Kim , M. Soto , M. Wagner , M.‐O. Turgeon , L. Fish , N. Erard , A L. Gable , A R. Maceli , S. Dickopf , E K. Papachristou , C S. D'Santos , L A. Carey , J E. Wilkinson , J. C. Harrell , C M. Perou , H. Goodarzi , G. Poulogiannis , G J. Hannon , Nature 2018, 554, 378. 29414946 10.1038/nature25465 PMC5898613

[102] G. Doglioni , J. Fernández‐García , S. Igelmann , P. Altea‐Manzano , A. Blomme , R. La Rovere , X.‐Z. Liu , Y. Liu , T. Tricot , M. Nobis , N. An , M. Leclercq , S. El Kharraz , P. Karras , Y.‐H. Hsieh , F A. Solari , L. Martins Nascentes Melo , G. Allies , A. Scopelliti , M. Rossi , I. Vermeire , D. Broekaert , A. M. Ferreira Campos , P. Neven , M. Maetens , K. Van Baelen , H. F. Alkan , M. Planque , G. Floris , A. Sickmann , et al., Nature 2025, 638, 244. 39743589 10.1038/s41586-024-08335-7 PMC7618879

[103] K G. Hicks , A A. Cluntun , H L. Schubert , S R. Hackett , J A. Berg , P G. Leonard , M A. Ajalla Aleixo , Y. Zhou , A J. Bott , S R. Salvatore , F. Chang , A. Blevins , P. Barta , S. Tilley , A. Leifer , A. Guzman , A. Arok , S. Fogarty , J M. Winter , H.‐C. Ahn , K N. Allen , S. Block , I A. Cardoso , J. Ding , I. Dreveny , W C. Gasper , Q. Ho , A. Matsuura , M J. Palladino , S. Prajapati , et al., Science 2023, 379, 996. 36893255 10.1126/science.abm3452 PMC10262665

[104] D. Martinez Molina , R. Jafari , M. Ignatushchenko , T. Seki , E. A. Larsson , C. Dan , L. Sreekumar , Y. Cao , P. Nordlund , Science 2013, 341, 84. 23828940 10.1126/science.1233606

[105] M M. Savitski , F B. M. Reinhard , H. Franken , T. Werner , M. F. Savitski , D. Eberhard , D. M. Molina , R. Jafari , R. B. Dovega , S. Klaeger , B. Kuster , P. Nordlund , M. Bantscheff , G. Drewes , Science 2014, 346, 1255784. 25278616 10.1126/science.1255784

[106] I. Piazza , K. Kochanowski , V. Cappelletti , T. Fuhrer , E. Noor , U. Sauer , P. Picotti , Cell 2018, 172, 358. 29307493 10.1016/j.cell.2017.12.006

[107] S. Schopper , A. Kahraman , P. Leuenberger , Y. Feng , I. Piazza , O. Muller , P. J. Boersema , P. Picotti , Nat. Protoc. 2017, 12, 2391. 29072706 10.1038/nprot.2017.100

[108] I. Piazza , N. Beaton , R. Bruderer , T. Knobloch , C. Barbisan , L. Chandat , A. Sudau , I. Siepe , O. Rinner , N. de Souza , P. Picotti , L. Reiter , Nat. Commun. 2020, 11, 4200. 32826910 10.1038/s41467-020-18071-x PMC7442650

[109] K. Li , S. Chen , K. Wang , Y. Wang , L. Xue , Y. Ye , Z. Fang , J. Lyu , H. Zhu , Y. Li , et al., Nat. Methods 2025, 22, 278. 39658593 10.1038/s41592-024-02553-7

[110] X. Gu , P. Jouandin , P. V. Lalgudi , R. Binari , M. L. Valenstein , M. A. Reid , A. E. Allen , N. Kamitaki , J. W. Locasale , N. Perrimon , D. M. Sabatini , Nature 2022, 608, 209. 35859173 10.1038/s41586-022-04960-2 PMC10112710

[111] M. Abu‐Remaileh , G. A. Wyant , C. Kim , N. N. Laqtom , M. Abbasi , S. H. Chan , E. Freinkman , D. M. Sabatini , Science 2017, 358, 807. 29074583 10.1126/science.aan6298 PMC5704967

[112] G. J. Ray , E. A. Boydston , E. Shortt , G. A. Wyant , S. Lourido , W. W. Chen , D. M. Sabatini , iScience 2020, 23, 101109. 32417403 10.1016/j.isci.2020.101109 PMC7254474

[113] C. H. Adelmann , A. K. Traunbauer , B. Chen , K. J. Condon , S. H. Chan , T. Kunchok , C. A. Lewis , D. M. Sabatini , Nature 2020, 588, 699. 33208952 10.1038/s41586-020-2937-x PMC7770032

[114] W. W. Chen , E. Freinkman , T. Wang , K. Birsoy , D. M. Sabatini , Cell 2016, 166, 1324. 27565352 10.1016/j.cell.2016.07.040 PMC5030821

[115] E. A. Siess , D. G. Brocks , O. H. Wieland , Hoppe Seylers Z. Physiol. Chem. 1978, 359, 785. 680639 10.1515/bchm2.1978.359.2.785

[116] R. Li , Y. Li , K. Jiang , L. Zhang , T. Li , A. Zhao , Z. Zhang , Y. Xia , K. Ge , Y. Chen , C. Wang , W. Tang , S. Liu , X. Lin , Y. Song , J. Mei , C. Xiao , A. Wang , Y. Zou , X. Li , X. Chen , Z. Ju , W. Jia , J. Loscalzo , Y. Sun , W. Fang , Y. Yang , Y. Zhao , Cell Metab. 2025, 37, 291. 39413790 10.1016/j.cmet.2024.09.011

[117] S. Sengupta , E. Giaime , S. Narayan , S. Hahm , J. Howell , D. O'Neill , G. P. Vlasuk , E. Saiah , Sci. Rep. 2019, 9, 4107. 30858438 10.1038/s41598-019-40693-5 PMC6412019

[118] C. Zhang , D. Liang , A. G. Ercan‐Sencicek , A S. Bulut , J. Cortes , I Q. Cheng , O. Henegariu , S. Nishimura , X. Wang , A. B. Peksen , Y. Takeo , C. Caglar , T T. Lam , M. N. Koroglu , A. Narayanan , F. Lopez‐Giraldez , D F. Miyagishima , K. Mishra‐Gorur , T. Barak , K. Yasuno , E. Z. Erson‐Omay , C. Yalcinkaya , G. Wang , S. Mane , H. Kaymakcalan , A. Guzel , A. O. Caglayan , B. Tuysuz , N. Sestan , M. Gunel , et al., Nature 2025, 638, 172. 39743596 10.1038/s41586-024-08341-9 PMC11798849

